# Physical Activity, Rumination, and Social Interaction Anxiety in Adolescents: A Cross-Sectional Moderated Network Analysis

**DOI:** 10.3390/bs16091461

**Published:** 2026-08-23

**Authors:** Pengfei Ji, Yongxin Wang, Jirui Qiu, Yansong Li, Xue Xia

**Affiliations:** 1 School of Physical Education, Qingdao University, Qingdao 266071, China; jipengfei@qdu.edu.cn (P.J.); 2715225984@qdu.edu.cn (Y.W.); qiujirui@qdu.edu.cn (J.Q.); 2 School of Social Development, University of Health and Rehabilitation Sciences, Qingdao 266113, China

**Keywords:** adolescents, rumination, social interaction anxiety, physical activity, moderated network model, brooding

## Abstract

(1) Background: Rumination is closely associated with adolescent social interaction anxiety, and physical activity has been linked to both repetitive negative thinking and anxiety-related symptoms. This study examined the network relationships between different dimensions of rumination and social interaction anxiety and explored the moderating role of physical activity level. (2) Methods: A total of 1194 adolescents aged 14–18 years (525 boys, 669 girls; M = 16.27, SD = 0.80) completed measures of rumination, social interaction anxiety, and physical activity. A moderated network model was estimated using the modnets package in R, and bootstrap procedures were used to assess network accuracy and stability. (3) Results: All rumination dimensions were positively connected, with the strongest association between symptom rumination and reflective pondering. Brooding showed the largest conditional association with social interaction anxiety among the three rumination dimensions and the highest expected influence. At higher physical activity levels, the estimated positive conditional association between symptom rumination and brooding was weaker, the association between reflective pondering and brooding was stronger, and the association between brooding and social interaction anxiety was weaker. (4) Conclusions: In the estimated network, brooding showed the highest expected influence and the largest conditional association with social interaction anxiety among the three rumination dimensions. Several estimated conditional associations involving brooding differed across physical activity levels. However, given the cross-sectional design and the limited stability of the interaction estimates, these moderation patterns should be regarded as exploratory. They reflect correlational differences in the estimated network across physical activity levels rather than evidence of a protective effect of physical activity.

## 1. Introduction

Social interaction anxiety refers to experiences of tension, anxiety, and avoidance in social or evaluative situations arising from concerns about negative evaluation, rejection, or embarrassing performance. Cognitive–behavioral models suggest that social interaction anxiety is associated not only with external social situations but also with individuals’ internal processing of social-evaluative information. Evidence suggests that individuals with higher levels of social interaction anxiety are more likely to focus their attention on their own performance and potential negative evaluation in social or evaluative situations, and they tend to process social-evaluative information in biased ways (Clark & Wells, 1995; Rapee & Heimberg, 1997). Further research has emphasized that self-focused attention, negative self-evaluation, and post-event processing contribute to the development and maintenance of social interaction anxiety, and related cognitive models have also been used to understand and intervene in adolescent social interaction anxiety (Hofmann, 2007; Leigh & Clark, 2018; Wong & Rapee, 2016). Foundational research in adolescent populations showed that higher social anxiety was associated with poorer peer acceptance, lower perceived social support, and difficulties in close friendships (La Greca & Lopez, 1998). Subsequent work further linked peer victimization and negative friendship experiences with higher social anxiety during adolescence (La Greca & Harrison, 2005). Therefore, understanding the cognitive characteristics and associative patterns related to adolescent social interaction anxiety from the perspective of cognitive processing has clear theoretical and practical significance.

Among the cognitive factors involved in the maintenance of negative emotions, rumination is an important variable. Within response styles theory, rumination is conceptualized as a repetitive mode of thinking in which individuals attend to their negative emotions, related symptoms, and the possible causes and consequences of these experiences. This processing style may prolong negative emotional states and weaken individuals’ ability to engage in effective problem-solving behaviors (Nolen-Hoeksema et al., 2008; Nolen-Hoeksema, 1991). This theory provides the basis for selecting rumination as the core cognitive variable in the present study.

It should be further noted that rumination is not a completely homogeneous psychological construct. Psychometric research on rumination has found that different components of rumination may have different psychological functions (Treynor et al., 2003). Repetitive thinking may lead to unconstructive consequences, but it may also have constructive functions under certain conditions, depending on the processing style and specific content of the thinking (Watkins, 2008).

Although symptom rumination and brooding are conceptually related, they emphasize different aspects of ruminative processing. Symptom rumination primarily concerns the content toward which attention is directed, including negative emotions, somatic symptoms, and their perceived causes and consequences. By contrast, brooding reflects a passive and evaluative processing mode characterized by recurrent comparison between one’s current situation and unattained standards. Reflective pondering involves more deliberate inward analysis that may be directed toward understanding a problem or identifying possible solutions. Thus, symptom rumination and brooding may share negative content while differing in the manner in which that content is processed (Treynor et al., 2003; Watkins, 2008; Nolen-Hoeksema et al., 2008).

This distinction may be particularly relevant to social interaction anxiety. Brooding involves sustained evaluative processing and difficulty disengaging from perceived discrepancies, which conceptually resembles the self-focused attention, negative self-evaluation, and post-event processing emphasized in cognitive models of social interaction anxiety. In social situations, this processing mode may involve repeated comparison between one’s perceived performance and internalized social standards, as well as continued review of possible mistakes or negative evaluations. Symptom rumination, in contrast, may reflect broader monitoring of emotional and somatic distress and may therefore show a less specific association with socially evaluative processing. On this basis, brooding may be expected to show a more prominent conditional association with social interaction anxiety, although the constructs remain correlated and some overlap in their measured content cannot be excluded.

From a broader transdiagnostic perspective, rumination can be situated within repetitive negative thinking (RNT), which is characterized by repetitive, negative, and relatively uncontrollable thought processes across a range of emotional problems (Ehring & Watkins, 2008; McEvoy et al., 2010; McKenney et al., 2023; Wahl et al., 2019). Meta-analytic evidence in nonclinical youth linked emotion-focused rumination with depressive symptoms (Rood et al., 2009), while longitudinal research indicated that rumination may partly account for the overlap between depressive and anxiety symptoms during adolescence (McLaughlin & Nolen-Hoeksema, 2011). Within social interaction anxiety, post-event processing can be understood as a more context-specific manifestation of repetitive negative thinking, involving repeated review of one’s social performance, possible mistakes, and anticipated negative evaluations (Brozovich & Heimberg, 2008; Edgar et al., 2024; Flynn & Yoon, 2025). Thus, RNT provides a broad transdiagnostic framework, whereas post-event processing refers to a socially situated repetitive-thinking pattern that is particularly relevant to social interaction anxiety. Examining different dimensions of rumination may therefore help clarify which forms of repetitive negative processing are most closely associated with adolescent social interaction anxiety.

In addition to cognitive factors, physical activity may also be a relevant behavioral factor in the relationship between rumination and social interaction anxiety. Regular physical activity has been closely linked to mental health (World Health Organization, 2020). Studies involving children and adolescents have linked higher physical activity with better mental health, lower anxiety symptoms, and fewer internalizing problems, including depression and anxiety (Biddle & Asare, 2011; Carter et al., 2021; Lubans et al., 2016; Singh et al., 2026). Findings from broader populations further indicate that physical activity is related to a reduced risk of incident anxiety and greater improvement in depression, anxiety, and psychological distress (Rebar et al., 2015; Schuch et al., 2019). These studies suggest that physical activity is not only a health-related behavior but also an important external behavioral factor for understanding adolescent mental health. For social interaction anxiety, the association between physical activity and anxiety experiences may involve emotional state, opportunities for social interaction, self-efficacy, and psychological regulatory resources.

Further research suggests that physical activity may be related not only to anxiety symptom levels but also to negative cognitive processing and emotion regulation. Reviews of intervention studies have reported associations between physical activity and reductions in repetitive negative thinking, particularly cognitive processes such as rumination and worry (S. Wang et al., 2025). Findings from adolescent samples further suggest that physical activity is related to lower repetitive negative thinking, and that positive affect and cognitive flexibility may partly explain this association (Xu et al., 2026). Higher habitual physical activity is also associated with a greater tendency to use adaptive cognitive reappraisal strategies, such as positive reinterpretation, when coping with stressful events (Perchtold-Stefan et al., 2020). Taken together, these studies suggest that physical activity may not only be associated with psychological symptom levels but may also be related to how individuals process negative information and regulate emotional responses.

Physical activity may also be more closely related to the processing characteristics of brooding than to the mere salience of negative symptoms. Previous findings linking physical activity with cognitive flexibility, adaptive reappraisal, and emotional recovery suggest that physical activity levels may be associated with individuals’ capacity to shift attention away from repetitive evaluative processing. Because brooding is characterized by cognitive perseveration and difficulty disengaging from negative self-evaluation, conditional associations involving brooding may be particularly likely to differ across physical activity levels. Nevertheless, this proposition concerns a theoretically plausible pattern rather than an established mechanism, and the present cross-sectional analysis was not designed to determine whether physical activity causally alters brooding.

In addition, some studies provide relatively direct evidence that physical activity may moderate the relationship between negative cognition and negative responses. For instance, the association of stressor-induced rumination with cortisol reactivity and recovery appears to vary by physical activity level (Puterman et al., 2011). Exercise has also been linked to weaker effects of rumination and emotion regulation difficulties on delayed emotional recovery (Bernstein & McNally, 2017, 2018). Thus, physical activity may be connected not only with rumination or anxiety levels, but also with the strength of associations between negative cognition and negative emotional or stress responses (Noetel et al., 2024; Singh et al., 2023). This rationale provides theoretical and empirical support for treating physical activity level as a moderator. Accordingly, physical activity was conceptualized as an external behavioral context within which cognitive–affective associations may differ. From this perspective, physical activity levels may characterize differences in the strength of the conditional associations between repetitive negative cognition and anxiety-related experiences rather than representing a constituent psychological node in the focal network.

However, several gaps remain in the existing literature. First, much of the previous research has examined rumination as an overall construct or focused on bivariate associations between rumination and anxiety, providing limited evidence regarding whether symptom rumination, brooding, and reflective pondering show distinct associations with social interaction anxiety when considered simultaneously. Second, physical activity has commonly been examined in relation to overall levels of rumination, anxiety, or psychological distress, whereas less is known about whether specific associations between rumination dimensions and social interaction anxiety differ across physical activity levels. Third, relatively little research has integrated these questions within a single multivariable framework in adolescent samples. Network approaches conceptualize psychological phenomena as systems of interconnected experiences or psychological components rather than solely as manifestations of a single underlying factor (Borsboom, 2017; Fried & Cramer, 2017). From this perspective, symptom rumination, brooding, reflective pondering, and social interaction anxiety can be examined as interconnected cognitive–emotional constructs. Moderated network models further allow the conditional association between two nodes to vary as a function of a third variable (Haslbeck et al., 2021). Accordingly, the present study extends previous research by differentiating the major dimensions of rumination, estimating their conditional associations with social interaction anxiety within a single network, and examining whether these edge-specific associations vary across physical activity levels. Given the cross-sectional design, this contribution concerns the structure of correlational associations rather than causal mechanisms.

Methodologically, the moderated network model was selected because it matched the edge-specific research question of the present study, rather than because it was assumed to be inherently superior to conventional analytical approaches. A conventional moderation-regression strategy would generally be organized as a set of outcome-specific models in which predictor and outcome roles are designated in advance. By contrast, the present study sought to estimate the full set of pairwise conditional associations among symptom rumination, brooding, reflective pondering, and social interaction anxiety within a single undirected system and to examine whether each specific association varied across the continuous physical activity moderator. Although structural equation modeling can be used to examine theory-driven moderation and latent-variable relationships, a directional structural model would require a prespecified ordering of the focal constructs that was not warranted by the cross-sectional design. Mediation analysis was also not aligned with the present research question because no indirect pathway or temporal sequence through physical activity was proposed. Accordingly, the moderated network model was used to estimate edge-specific conditional associations while accounting for the remaining modeled nodes and to examine whether these associations differed across physical activity levels (Haslbeck et al., 2021).

Treating physical activity as a moderator rather than as a network node reflects the specific research question of the present study. If physical activity were included as a node, the model would estimate its direct conditional associations with symptom rumination, brooding, reflective pondering, and social interaction anxiety. By contrast, specifying physical activity as a moderator allows the model to examine whether the conditional associations among these focal psychological constructs differ across physical activity levels. This approach enables the identification of edge-specific moderated associations, although it does not estimate the direct conditional relationships between physical activity and each psychological construct.

To address these gaps, the present study constructed a rumination–social interaction anxiety moderated network model with physical activity level as the moderator. The model was informed by response styles theory, cognitive–behavioral accounts of social interaction anxiety, the transdiagnostic perspective of repetitive negative thinking, research on physical activity and cognitive–emotional regulation, and psychopathological network theory. Specifically, symptom rumination, brooding, and reflective pondering were treated as cognitive nodes within the rumination system, social interaction anxiety was treated as an emotional node, and physical activity level was examined as a moderator of the connections among these nodes. Based on the theoretical distinction among rumination dimensions and the conceptual correspondence between brooding and the self-focused evaluative processing emphasized in cognitive models of social interaction anxiety, we hypothesized that brooding would show a more prominent positive conditional association with social interaction anxiety than symptom rumination and reflective pondering (H1). Because previous evidence was insufficient to specify the precise pattern and direction of the remaining edge-level and moderation effects, the study was additionally guided by the following research questions: RQ1: What is the overall pattern of estimated conditional associations among symptom rumination, brooding, reflective pondering, and social interaction anxiety? RQ2: Which conditional associations among these focal psychological constructs vary across physical activity levels, and in what directions? RQ3: Which focal node shows the greatest signed connectivity as indexed by expected influence?

## 2. Materials and Methods

### 2.1. Participants

The study used a cross-sectional design based on questionnaire data. Participants were recruited through convenience sampling from one public secondary school in Shandong Province, China, which was selected through an existing collaborative relationship with the research team. Data were collected in December 2025 using an online questionnaire administered through Wenjuanxing. Before questionnaire administration, teachers and researchers explained the study objectives and procedures to the students. Participation was voluntary.

An initial total of 1400 adolescents participated in the survey. During the data cleaning process, 206 questionnaires were excluded due to missing values or failure to pass attention check items. Consequently, a final sample of 1194 valid questionnaires was retained, yielding an effective response rate of 85.3%. The participants were aged 14–18 years (M = 16.27, SD = 0.80) and included 525 boys and 669 girls.

### 2.2. Measures

#### 2.2.1. Chinese Version of the Ruminative Responses Scale

Rumination was measured with the Chinese Version of the Ruminative Responses Scale (RRS-CV). This scale is used to assess individuals’ tendency to attend to their emotions, symptoms, and the possible causes of these experiences in negative emotional states. The RRS-CV includes 22 items scored on a 4-point response scale, with higher scores reflecting stronger rumination tendencies.

Based on the dimensional structure of the scale, scores were calculated separately for symptom rumination, brooding, and reflective pondering. Symptom rumination reflects recurrent attention to negative emotional and somatic symptoms and their perceived causes and consequences. Brooding reflects a passive, abstract, and evaluative mode of thinking, including recurrent comparison between one’s current situation and unattained standards. Reflective pondering reflects more deliberate inward analysis aimed at understanding problems and considering possible solutions (Treynor et al., 2003). In this study, symptom rumination, brooding, and reflective pondering were used as cognitive nodes in the moderated network model and were labeled A1, A2, and A3, respectively.

#### 2.2.2. Interaction Anxiousness Scale

The Interaction Anxiousness Scale (IAS) was used to assess social interaction anxiety. This scale is mainly used to assess subjective tension, anxiety, and discomfort in social interaction situations. The IAS contains 15 items scored on a 5-point scale; items 3, 6, 10, and 15 are reverse-scored. Higher total scores reflect greater anxiety in social interaction situations. Leary and Kowalski (1993) provided evidence for the construct and criterion-related validity of the IAS. Its discriminative validity for screening social anxiety was also supported in a Chinese college student sample (Cao et al., 2016).

In this study, the IAS total score was used as an indicator of social interaction anxiety and was treated as the social interaction anxiety node in the moderated network model, labeled B1.

#### 2.2.3. Physical Activity Rating Scale-3

Physical activity level was measured using Liang’s revised Physical Activity Rating Scale-3 (PARS-3). The PARS-3 estimates exercise volume based on intensity, duration, and frequency, calculated as intensity × duration × frequency. Intensity and frequency are scored from 1 to 5, while duration is scored from 0 to 4, ranging from 0 to 100 (Zhou et al., 2025). Higher scores represent greater physical activity levels.

In this study, the PARS-3 total exercise-volume score was used as a continuous moderator in the moderated network model and was labeled M.

The internal consistency of the main measures was examined in the current sample. The Cronbach’s α coefficients were 0.979 for the total RRS-CV, 0.964 for symptom rumination, 0.909 for brooding, 0.923 for reflective pondering, and 0.954 for the IAS. McDonald’s ω coefficients were 0.979 for the total RRS-CV, 0.964 for symptom rumination, 0.910 for brooding, 0.925 for reflective pondering, and 0.955 for the IAS. Overall, both reliability indices indicated high internal consistency. However, coefficients exceeding 0.95 for several measures may also reflect very high item homogeneity or partially overlapping item content and should not be interpreted solely as evidence of superior measurement quality.

### 2.3. Statistical Analysis

Statistical analyses were conducted using R 4.5.2. First, the study variables were organized, and scores for symptom rumination, brooding, reflective pondering, social interaction anxiety, and physical activity level were calculated.

Subsequently, the modnets package was used to construct a moderated network model with physical activity level as the moderator. In the model, symptom rumination, brooding, reflective pondering, and social interaction anxiety were specified as network nodes, while the PARS-3 physical activity level was specified as the moderator. The model was used to test whether physical activity level moderated the conditional associations among the rumination dimensions and between each rumination dimension and social interaction anxiety. The moderated network model was selected to estimate the full set of pairwise conditional associations among the four focal constructs within a single undirected system and to test whether each edge varied across the continuous physical activity moderator. This model specification was aligned with the edge-specific research question and did not impose directional or mediational pathways among the cross-sectionally measured constructs (Haslbeck et al., 2021).

Model estimation was performed with the fitNetwork function, specifying the OR rule for edge selection. This function conducts nodewise regression through a series of independent univariate regression models for each variable. During primary-model estimation, variables were mean-centered but not z-standardized. In this study, a conditional association refers to the regression-based association between two nodes after accounting for the remaining modeled nodes. Accordingly, the pairwise edge weights reflect estimated conditional associations under mean-centered conditions, whereas interaction terms indicate whether these conditional associations vary across physical activity levels.

The estimated network was visualized using qgraph; nodes corresponded to study variables, edges represented conditional associations, edge weights indicated association strength, and blue and red edges denoted positive and negative associations, respectively (Epskamp et al., 2012).

For network edges showing potential moderation, the condPlot function was used to display the estimated conditional association across observed levels of physical activity. The shaded regions represent pointwise 95% confidence intervals for the estimated conditional associations. For each edge, the bootstrap 95% confidence interval for the difference between the estimated conditional associations at the maximum and minimum observed levels of physical activity (the max–min difference) was used to assess nominal support for variation across physical activity levels. This interval should not be interpreted as the confidence interval of the regression interaction coefficient itself.

For associations showing nominal support for moderation, Johnson–Neyman analyses were additionally conducted to identify the observed physical activity values at which the pointwise 95% confidence interval of the estimated conditional association excluded zero. Johnson–Neyman thresholds are reported on the original physical activity scale (0–100).

To examine whether the primary findings were robust after accounting for demographic differences, an additional covariate-adjusted moderated network model was estimated using the same specifications as the primary model. Age was included as a continuous covariate, and sex was included as a binary covariate (1 = boys, 2 = girls). Age and sex were entered into the nodewise regression models but were not treated as network nodes or included in the expected influence analysis. This analysis was conducted as a sensitivity analysis rather than as a formal test of network invariance across age or sex groups.

To examine whether the pattern of pairwise conditional associations was sensitive to differences in measurement scale, a standardized-node sensitivity analysis was conducted. Symptom rumination (A1), brooding (A2), reflective pondering (A3), social interaction anxiety (B1), and physical activity (M) were z-standardized, and the moderated network was re-estimated using the same OR rule. Pairwise conditional associations at the mean physical activity level were then compared with those from the primary raw-score model.

To evaluate the signed overall connectivity of each node, expected influence (EI) was calculated by summing the positive and negative edge weights connected to that node. EI was selected because the estimated network contained both positive and negative conditional associations, and EI preserves the signs of these associations, whereas strength centrality is based on the absolute magnitudes of edge weights. EI was interpreted as an index of signed overall connectivity and not as evidence of directional or causal influence. Betweenness and closeness were not examined because the present network comprised only four densely connected aggregate constructs, limiting the additional interpretive value of path-based centrality indices. Physical activity level was treated as the moderator and was not included in the node centrality analysis. Given the limitations of centrality indices in psychological networks, EI was interpreted cautiously and in conjunction with the bootstrap centrality-difference test (Bringmann et al., 2019; Robinaugh et al., 2016).

A total of 1000 nonparametric bootstrap resamples were used to assess the accuracy and stability of edge-weight and expected-influence estimates and to test differences among these estimates. Accuracy tests were used to evaluate the confidence intervals of edge-weight and EI estimates. Bootstrap difference tests were used to compare pairwise edge weights and node expected-influence values. Differences whose nominal 95% bootstrap confidence intervals did not include zero were regarded as nominally supported. No adjustment for multiple comparisons was applied because these tests were used as secondary exploratory analyses to contextualize the relative pattern of the network estimates rather than as confirmatory tests of a prespecified family of hypotheses. Case-dropping bootstrap analysis was used to evaluate stability, with the correlation stability coefficient serving as the criterion. In line with previous methodological recommendations, coefficients above 0.25 were considered acceptable, whereas coefficients above 0.50 were interpreted as indicating good stability (Costenbader & Valente, 2003; Epskamp et al., 2018).

## 3. Results

### 3.1. Descriptive Statistics of Network Variables

Descriptive statistics of the five variables included in the moderated network model are presented in Table 1. The means, standard deviations, and observed ranges of symptom rumination (A1), brooding (A2), reflective pondering (A3), social interaction anxiety (B1), and physical activity level (M) are provided to characterize the distribution of the network variables.

### 3.2. Overall Structure of the Moderated Network Model

**Figure 1:**
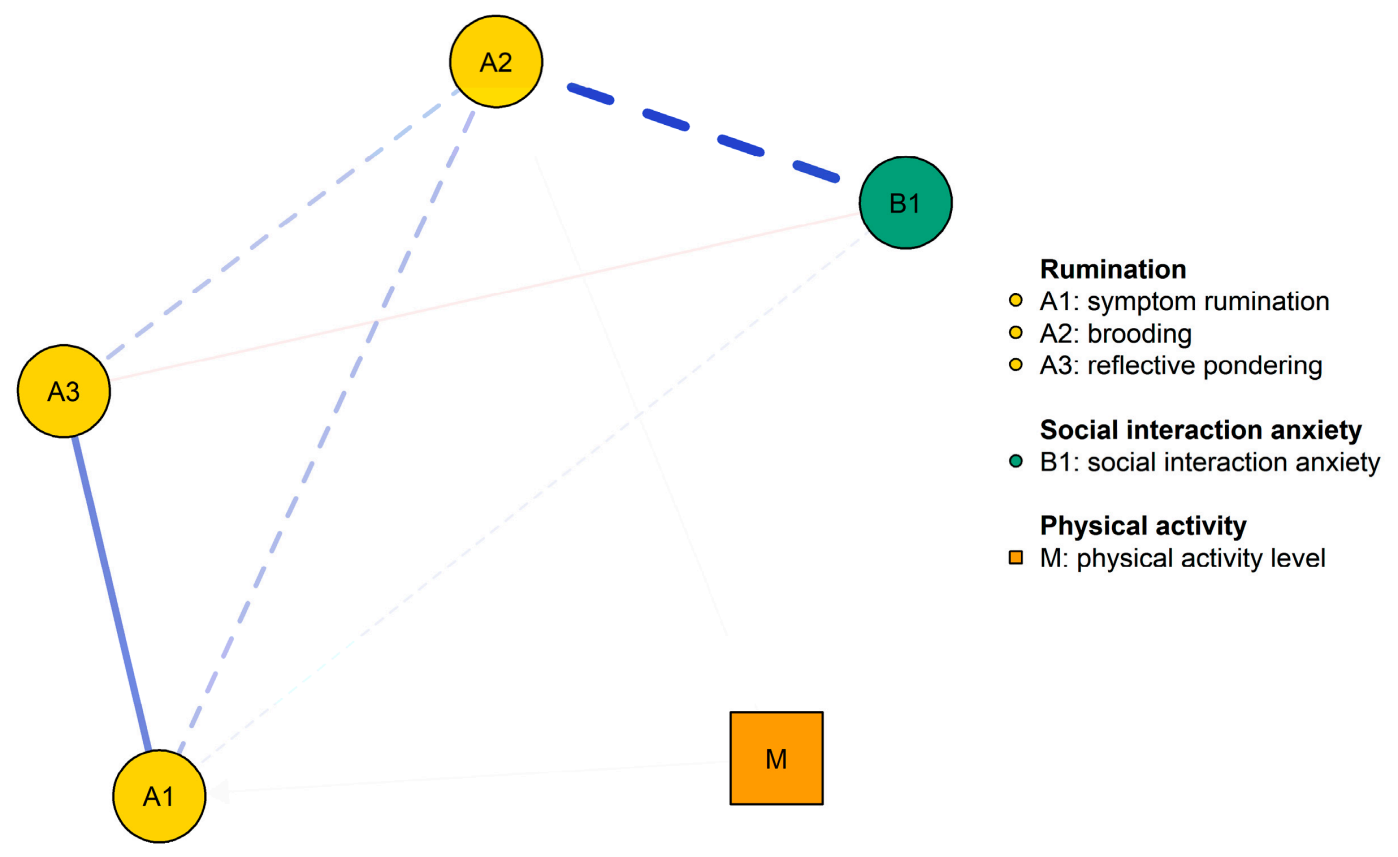
Estimated moderated network of symptom rumination (A1), brooding (A2), reflective pondering (A3), and social interaction anxiety (B1), with physical activity level (M) specified as an external moderator. Solid edges represent estimated pairwise conditional associations, whereas dashed edges indicate pairwise associations for which a moderation term involving M was retained in the estimated model. Blue and red edges indicate positive and negative estimates, respectively, and edge width reflects the absolute magnitude of the estimate. M is displayed as a square because it was modeled as a moderator rather than as a focal psychological node.

The network results showed that all dimensions of rumination were positively connected. Among them, the edge between symptom rumination and reflective pondering showed the highest edge weight (edge weight = 0.82). Symptom rumination was also clearly and positively connected with brooding (edge weight = 0.46), whereas the connection between brooding and reflective pondering was relatively weaker but remained positive (edge weight = 0.39). The overall structure of the estimated moderated network is presented in Figure 1.

Among the associations between rumination and social interaction anxiety, the estimated edge weight between brooding and social interaction anxiety (A2–B1) was the highest (edge weight = 1.20). The subsequent edge-difference test indicated that this edge was larger than the other associations between the rumination dimensions and social interaction anxiety. This comparison is limited to these three associations and should not be interpreted as a ranking of all network connections. In contrast, the conditional association between symptom rumination and social interaction anxiety was weakly positive (A1–B1, edge weight = 0.09), whereas the conditional association between reflective pondering and social interaction anxiety was weakly negative (A3–B1, edge weight = −0.11). The complete pairwise edge-weight estimates and their bootstrap 95% confidence intervals are presented in Table 2.

### 3.3. Moderated Conditional Associations Across Physical Activity Levels

Marginal-effect analyses identified three moderated conditional associations with nominal statistical support across physical activity levels. The 95% confidence intervals reported below are bootstrap confidence intervals for the max–min differences in estimated conditional associations. Johnson–Neyman analyses were additionally used to identify the observed physical activity ranges in which the pointwise 95% confidence interval of each estimated conditional association excluded zero.

**Figure 2:**
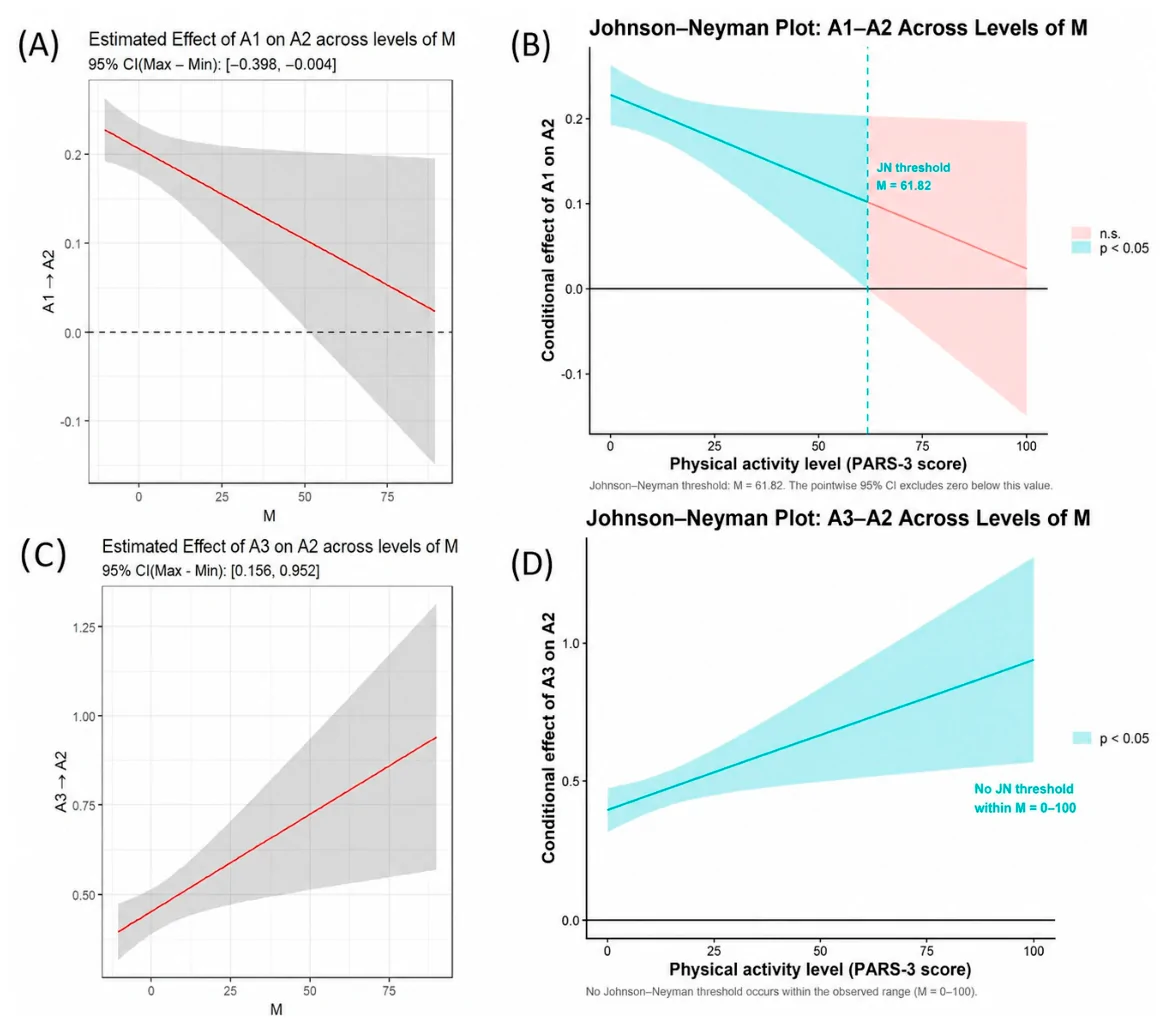
Moderated conditional associations involving brooding across physical activity levels. (**A**) Marginal-effect plot for the symptom rumination–brooding association (A1–A2). (**B**) Johnson–Neyman plot for A1–A2. (**C**) Marginal-effect plot for the reflective pondering–brooding association (A3–A2). (**D**) Johnson–Neyman plot for A3–A2. In panels (**A**,**C**), solid lines represent estimated conditional associations and shaded regions represent pointwise 95% confidence intervals; the bootstrap 95% confidence interval displayed above each panel represents the max–min difference across the observed physical-activity range. In panels (**B**,**D**), turquoise indicates physical activity levels at which the pointwise 95% confidence interval excludes zero (*p* < 0.05), whereas pink indicates levels at which it includes zero. The vertical dashed line denotes the Johnson–Neyman threshold, where applicable.

First, a moderated conditional association was observed between symptom rumination and brooding, as shown in Figure 2A. The estimated positive conditional association between symptom rumination and brooding was lower at higher than at lower physical activity levels. The bootstrap 95% confidence interval for the max–min difference in this estimated conditional association was [−0.398, −0.004]. Johnson–Neyman analysis indicated that the estimated positive conditional association was distinguishable from zero when physical activity was below 61.82; at physical activity levels of 61.82 or higher, its pointwise 95% confidence interval included zero.

Second, a moderated conditional association was observed between reflective pondering and brooding, as shown in Figure 2C. The estimated positive conditional association between reflective pondering and brooding was higher at higher than at lower physical activity levels. The bootstrap 95% confidence interval for the max–min difference in this estimated conditional association was [0.156, 0.952]. Johnson–Neyman analysis identified no threshold within the observed physical activity range (0–100): the estimated positive conditional association remained distinguishable from zero throughout this range.

**Figure 3:**
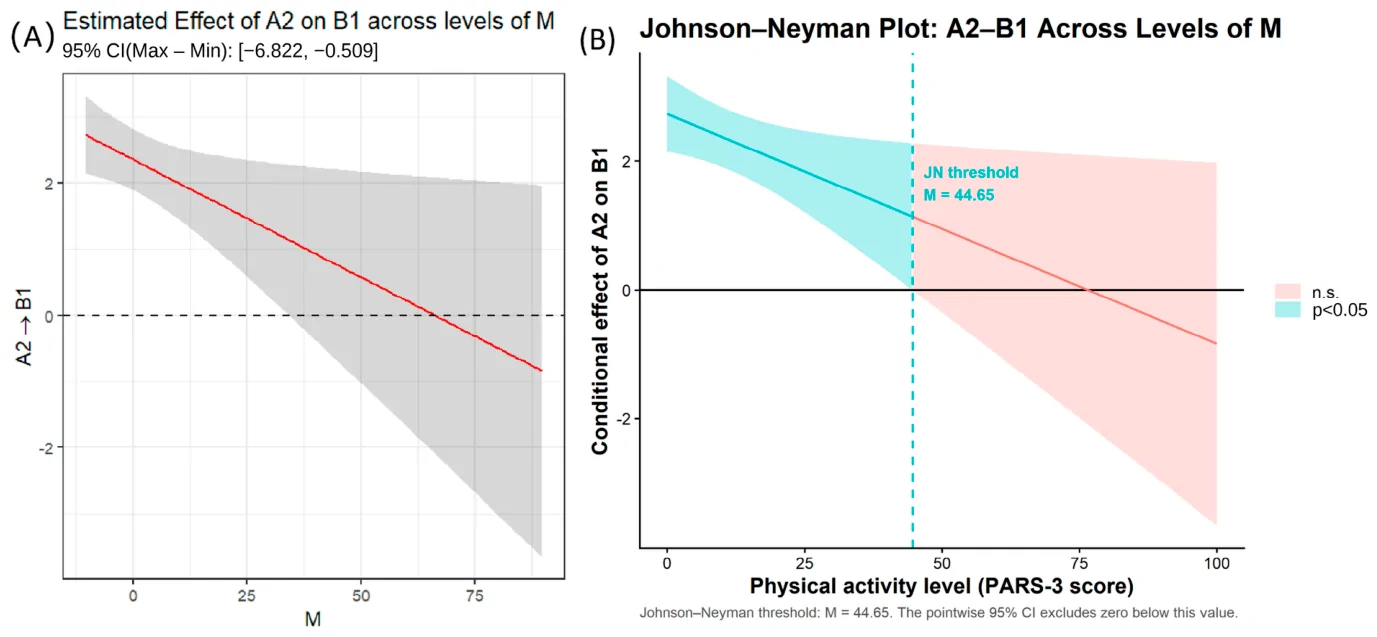
Moderated conditional association between brooding and social interaction anxiety across physical activity levels. (**A**) Marginal-effect plot for the brooding–social interaction anxiety association (A2–B1). The solid line represents the estimated conditional association, and the shaded region represents its pointwise 95% confidence interval. The bootstrap 95% confidence interval displayed above the panel represents the max–min difference in the estimated conditional association across the observed physical-activity range. (**B**) Johnson–Neyman plot for A2–B1. Turquoise indicates physical activity levels at which the pointwise 95% confidence interval excludes zero (*p* < 0.05), whereas pink indicates levels at which it includes zero. The vertical dashed line denotes the Johnson–Neyman threshold (M = 44.65).

Third, a statistically supported moderated conditional association was observed between brooding and social interaction anxiety, as shown in Figure 3A. At higher, relative to lower, levels of physical activity, the estimated conditional association between brooding and social interaction anxiety was lower. The bootstrap 95% confidence interval for the difference in estimated conditional effects between the maximum and minimum observed levels of physical activity was [−6.822, −0.509]. As shown in Figure 3B, the Johnson–Neyman analysis indicated that the estimated positive conditional association between brooding (A2) and social interaction anxiety (B1) was statistically distinguishable from zero when physical activity level (M) was at or below 44.65 (observed range: 0–100); above this value, the 95% confidence interval included zero.

Except for the three paths of symptom rumination–brooding, reflective pondering–brooding, and brooding–social interaction anxiety, the moderating effects on the symptom rumination–reflective pondering, symptom rumination–social interaction anxiety, and reflective pondering–social interaction anxiety paths did not reach statistical significance. Therefore, subsequent interpretation mainly focused on the three paths of symptom rumination–brooding, reflective pondering–brooding, and brooding–social interaction anxiety.

### 3.4. Sensitivity Analyses

An additional moderated network model was estimated with age and sex included as covariates. As shown in Table S1, Panel A, the pairwise conditional associations at the mean physical activity level were broadly similar before and after adjustment for age and sex. The positive associations between symptom rumination and brooding (0.457 vs. 0.457), symptom rumination and reflective pondering (0.821 vs. 0.823), brooding and reflective pondering (0.386 vs. 0.386), and brooding and social interaction anxiety (1.197 vs. 1.141) remained comparable. The symptom rumination–social interaction anxiety association remained small, and its 95% confidence interval included zero in both models (0.094 vs. 0.080). The weak negative association between reflective pondering and social interaction anxiety attenuated toward zero after adjustment (−0.108 vs. −0.014), with 95% confidence intervals including zero in both models. For the three focal moderated associations, the max–min differences in the estimated conditional associations were similar before and after adjustment for age and sex. For the symptom rumination–brooding association, the max–min difference changed from −0.205 in the primary model to −0.204 in the adjusted model. For the reflective pondering–brooding association, the corresponding difference changed from 0.544 to 0.545. For the brooding–social interaction anxiety association, the max–min difference changed from −3.571 to −3.301. Thus, adjustment for age and sex did not materially alter the direction or magnitude of the three focal moderated associations across the observed physical activity range (Table S1, Panel B). In addition, as shown in Table S2, z-standardization yielded the same signs for all six pairwise conditional associations as the primary raw-score model. Overall, adjustment for age and sex did not materially alter the pattern of pairwise conditional associations at the mean physical activity level or the max–min differences for the three focal moderated associations, and z-standardization yielded the same signs for all six pairwise conditional associations.

### 3.5. Centrality Analysis

Node signed connectivity within the network was evaluated using expected influence. Brooding (A2) showed the highest EI value (EI = 2.040). The EI values for symptom rumination (A1), social interaction anxiety (B1), and reflective pondering (A3) were 1.373, 1.184, and 1.099, respectively. Overall, brooding showed the greatest signed connectivity in the present network. Expected influence estimates are visualized in Supplementary Figure S1.

### 3.6. Edge and Centrality Difference Tests

**Figure 4:**
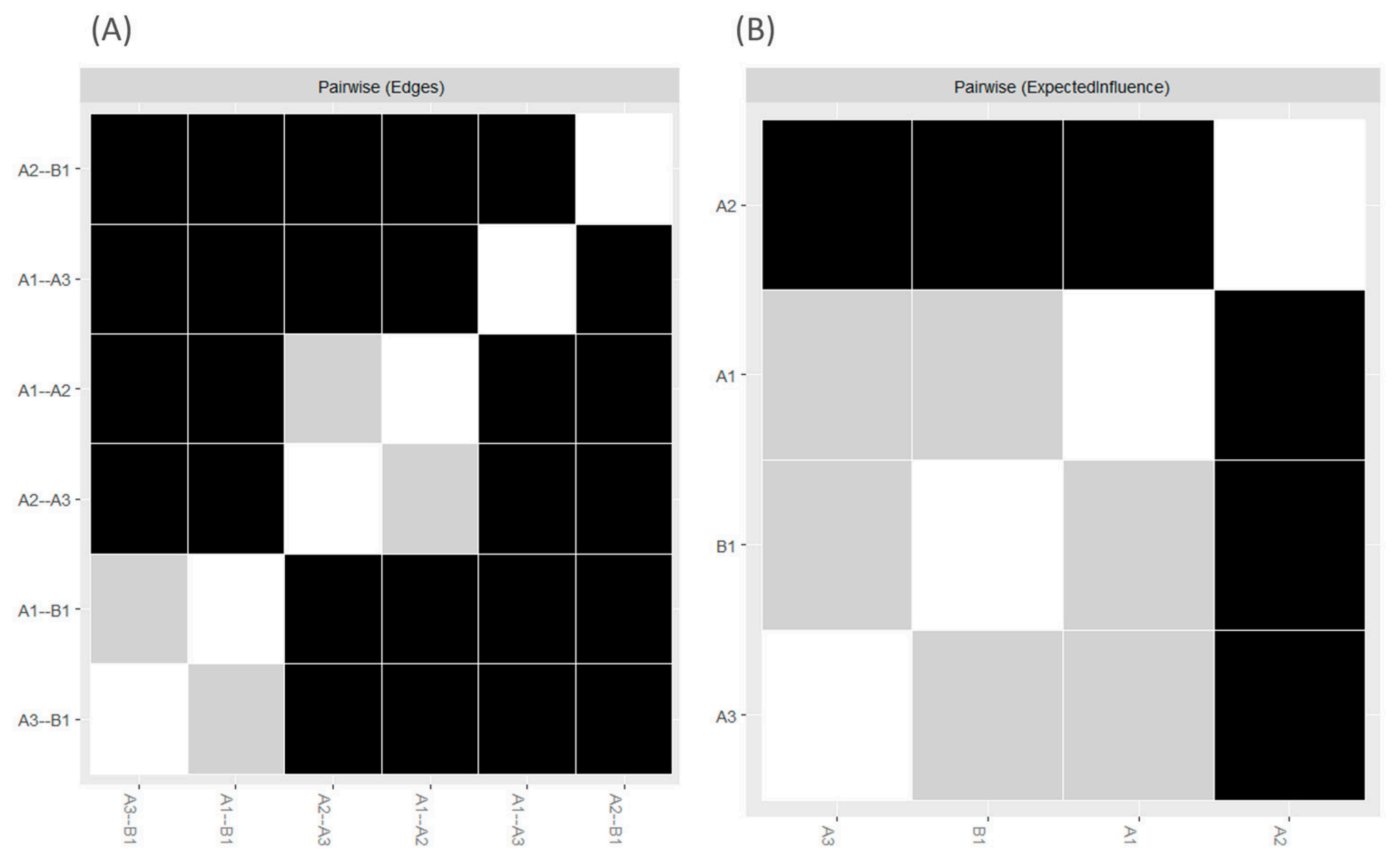
Bootstrap difference tests for pairwise edge weights and expected influence values. (**A**) Pairwise edge-weight difference test. Each cell represents a comparison between two estimated pairwise edge weights. (**B**) Expected influence difference test. Each cell represents a comparison between the expected influence values of two nodes. Black cells indicate statistically significant differences, whereas gray cells indicate nonsignificant differences. A difference was considered statistically supported when the bootstrap 95% confidence interval for the difference between the two estimates did not include zero. A1 = symptom rumination; A2 = brooding; A3 = reflective pondering; B1 = social interaction anxiety.

As shown in Figure 4A, the edge-difference test showed that the brooding–social interaction anxiety edge (A2–B1) differed significantly from the other pairwise edges. Combined with the edge-weight estimates, A2–B1 was the largest association between the rumination dimensions and social interaction anxiety. The symptom rumination–reflective pondering edge (A1–A3) also differed significantly from most other edges, suggesting that it was a relatively prominent connection within rumination. In contrast, some other pairwise edge differences did not reach statistical significance.

As shown in Figure 4B, the centrality-difference test showed that the expected influence of brooding (A2) was significantly higher than that of symptom rumination (A1), social interaction anxiety (B1), and reflective pondering (A3). In contrast, the differences in expected influence among symptom rumination, social interaction anxiety, and reflective pondering did not reach statistical significance. These results indicate that brooding showed significantly greater signed connectivity than the other nodes in the present network.

### 3.7. Network Accuracy and Stability Tests

The sample estimates of pairwise edge weights were generally close to the bootstrap means, with relatively narrow confidence intervals, indicating good accuracy of the pairwise edge-weight estimates. The expected influence estimates also showed good consistency, with the expected influence of brooding remaining consistently high. The bootstrap accuracy plots are provided in Supplementary Figure S2.

Case-dropping bootstrap results showed CS coefficients of 0.75 for the pairwise edge estimates and 0.67 for expected influence, indicating good stability of the pairwise network estimates. Interaction edges and interaction expected influence both had CS coefficients of 0.28. Although these values were slightly above the minimum reference value of 0.25, they indicate that the interaction-related estimates may be sensitive to case deletion. Accordingly, the moderation findings should be regarded as preliminary and exploratory and require independent replication. The corresponding case-dropping bootstrap stability plots are provided in Supplementary Figure S3.

## 4. Discussion

The present study estimated a moderated network of rumination dimensions and social interaction anxiety. Brooding showed the highest expected influence and the largest positive conditional association with social interaction anxiety among the three rumination dimensions. Several estimated conditional associations also differed across physical activity levels. However, because all variables were assessed concurrently, these findings represent cross-sectional moderated conditional associations and do not indicate that physical activity changed, weakened, or strengthened the network connections. Moreover, given the limited stability of the interaction-related estimates, the moderation patterns should be regarded as exploratory.

First, the central position of brooding in the network indicates that the relationships between different dimensions of rumination and social interaction anxiety are not identical. Rumination has been conceptualized as a multidimensional rather than a unitary construct, with components such as brooding and reflective pondering showing differential relationships with psychological symptoms. Repetitive thinking may also have constructive or unconstructive consequences depending on its processing style (Treynor et al., 2003; Watkins, 2008). From a network perspective, the current results showed that, among symptom rumination, brooding, and reflective pondering, brooding showed the most prominent connection with social interaction anxiety and had the highest expected influence. Among the three associations between rumination dimensions and social interaction anxiety, the brooding–social interaction anxiety association was the largest. Brooding also showed the highest expected influence.

The prominence of brooding may reflect its evaluative processing style rather than merely greater attention to negative symptoms. Symptom rumination primarily concerns recurrent attention to negative emotional and symptom-related experiences and their perceived causes and consequences, whereas brooding involves a passive, abstract, and evaluative mode of processing characterized by recurrent comparison between one’s current situation and unattained standards (Treynor et al., 2003; Nolen-Hoeksema et al., 2008; Watkins, 2008). In socially evaluative contexts, adolescents may repeatedly compare their perceived performance with internalized social standards, review possible mistakes, and reconstruct possible negative evaluations by others. These processes are conceptually consistent with the self-focused attention, negative self-evaluation, and post-event processing emphasized in cognitive models of social interaction anxiety (Clark & Wells, 1995; Rapee & Heimberg, 1997; Hofmann, 2007; Brozovich & Heimberg, 2008; Edgar et al., 2024; Flynn & Yoon, 2025). This theoretical correspondence may help explain why brooding showed a more prominent conditional association with social interaction anxiety after the remaining rumination dimensions were taken into account. Nevertheless, symptom rumination and brooding remain conceptually related, and some overlap in their measured content cannot be excluded. Because the present study used a cross-sectional network design, this interpretation does not imply that brooding causes social interaction anxiety.

Second, the moderating role of physical activity level in the brooding–social interaction anxiety conditional association was another key result. Exercise psychology research has commonly linked physical activity with lower anxiety and reduced negative cognitive processing. Studies involving children and adolescents have shown that physical activity may improve anxiety- and depression-related symptoms (Biddle & Asare, 2011; Carter et al., 2021; Singh et al., 2026), while reviews involving adults and broader populations indicate potential benefits for anxiety, depressive symptoms, and psychological distress (Rebar et al., 2015; Schuch et al., 2019; Singh et al., 2023; Stubbs et al., 2017). More direct evidence comes from studies treating exercise as a moderator. In the study by Puterman et al. (2011), the association between stressor-induced rumination and cortisol responses varied by physical activity level. Bernstein and McNally (2018) also found that exercise attenuated the effects of rumination and emotion-regulation difficulties on delayed emotional recovery. The shared implication of these studies is that physical activity may be linked not only to lower negative cognition, but also to weaker links between negative cognition and negative emotional or stress responses.

However, the available evidence is not entirely consistent. A recent meta-analysis of exercise interventions in adolescents found that the pooled effect on anxiety was not statistically significant and that between-study heterogeneity was substantial (T. Wang et al., 2024). Similarly, a school-based cluster randomized trial found no overall improvement in anxiety, although favorable effects were observed among adolescents with moderate baseline symptoms (da Silva et al., 2025). These mixed findings suggest that the association between physical activity and anxiety-related outcomes may vary according to baseline symptom severity, activity characteristics, intervention context, and outcome measurement. Accordingly, the present cross-sectional moderation pattern should not be interpreted as evidence that physical activity has a uniform protective effect on anxiety.

In the present network, the estimated positive conditional association between brooding and social interaction anxiety was weaker at higher physical activity levels. The Johnson–Neyman analysis indicated that the estimated positive conditional association was statistically distinguishable from zero only when physical activity level was below approximately 44.65. This value lies just above the conventional cutoff for high physical activity on the PARS-3 ≥ 43. This pattern indicates a cross-sectional difference in the estimated association across physical activity levels; it does not demonstrate that physical activity weakened, buffered, or reduced the connection. Because the variables were measured simultaneously, the temporal ordering of this pattern cannot be determined. Adolescents experiencing lower brooding or social interaction anxiety may be more likely to engage in physical activity, and the observed pattern may also reflect unmeasured characteristics associated with both physical activity and psychological functioning. Potential confounding factors include baseline affect, depressive and broader internalizing symptoms, peer support, family environment, socioeconomic circumstances, physical health, and access to organized physical activity opportunities. These variables were not included in the present model and may partly account for the observed moderated conditional association.

The estimated symptom rumination–brooding conditional association was weaker, whereas the estimated reflective pondering–brooding conditional association was stronger, at higher physical activity levels. These findings indicate cross-sectional differences in the estimated internal structure of rumination across physical activity levels rather than changes caused by physical activity. Moreover, the stronger reflective pondering–brooding association should not necessarily be interpreted as an adaptive cognitive pattern. It may reflect closer coupling between reflective analysis and persistent negative thinking or greater cognitive perseveration. The functional meaning of this finding therefore remains uncertain and requires replication.

The concentration of the estimated moderation patterns in brooding-related associations may be consistent with the possibility that physical activity level is more closely related to cognitive disengagement, emotional recovery, and flexible regulation than to the monitoring of negative symptoms itself. Because brooding is characterized by passive evaluative processing, cognitive perseveration, and difficulty disengaging from negative self-evaluation, its conditional associations may be particularly likely to differ across physical activity levels (Bernstein & McNally, 2017, 2018; Perchtold-Stefan et al., 2020). However, the present study did not directly assess attentional disengagement, cognitive flexibility, or emotion-regulation processes. Moreover, the interaction-related estimates showed limited stability. Therefore, this explanation should be regarded as a tentative theoretical interpretation rather than evidence that physical activity modified the underlying cognitive processes.

From the perspective of psychopathological network theory, psychological problems can be understood as networks formed by interactions among multiple symptoms or psychological components, instead of being viewed as expressions of a single underlying latent variable (Borsboom, 2017; Fried & Cramer, 2017). The present study treated different dimensions of rumination and social interaction anxiety as network nodes and further examined the moderating role of physical activity level in network connections. This is consistent with the methodological logic of moderated network models, which propose that relationships between nodes may be influenced by a third variable (Haslbeck et al., 2021). The estimated moderation patterns were concentrated primarily in brooding-related edges. This suggests that brooding-related conditional associations may provide a useful starting point for hypothesis generation in future replication studies. However, this concentration does not establish that physical activity causally modified these connections or that brooding should be regarded as a confirmed intervention target. From the perspective of bridge-node research in networks, examining highly connected nodes may help characterize associations among different psychological components (Jones et al., 2021).

The present findings offer three cautious theoretical contributions. First, at the cognitive level, they refine multidimensional accounts of rumination by suggesting that brooding, rather than rumination as a unitary construct, may be particularly relevant to the self-focused evaluative processing and post-event processing associated with adolescent social interaction anxiety. Second, at the behavioral level, physical activity was conceptualized not as a direct therapeutic factor or a constituent psychological node, but as an external behavioral context across which specific cognitive–affective associations may differ. This perspective extends the focus from whether physical activity is associated with overall symptom levels to whether the conditional associations among rumination dimensions and social interaction anxiety vary across physical activity levels. Third, at the network level, the findings suggest that heterogeneity in the rumination–social interaction anxiety network may be concentrated in specific brooding-related edges rather than distributed uniformly across the network. However, because the study was cross-sectional and the interaction-related estimates showed limited stability, these contributions should be regarded as provisional refinements of existing cognitive, behavioral, and network-based models rather than evidence of causal mechanisms.

The present study has implications primarily for future research. Focusing only on overall rumination may be insufficient, and the present findings suggest that brooding-related conditional associations may be a useful starting point for hypothesis generation in future longitudinal and experimental studies. However, the present results do not establish that increasing physical activity would reduce brooding, social interaction anxiety, or the association between them. Future studies should examine whether prospectively measured or experimentally manipulated physical activity is followed by changes in these cognitive–affective associations and whether such patterns differ according to activity type, dose, social context, and degree of structure.

The present study has several strengths. It differentiated symptom rumination, brooding, and reflective pondering rather than treating rumination as a unitary construct, thereby allowing their distinct conditional associations with social interaction anxiety to be examined. The moderated network approach further enabled the simultaneous estimation of associations among the focal constructs and the identification of edge-specific differences across continuous physical activity levels. In addition, the relatively large adolescent sample, bootstrap evaluations of estimation accuracy and stability, and age- and sex-adjusted sensitivity analysis increased the methodological transparency of the findings. Nevertheless, these strengths do not eliminate the limitations associated with the cross-sectional and self-report design or the limited stability of the interaction estimates.

### Limitations and Future Directions

Several limitations should be considered. First, the cross-sectional design precludes conclusions regarding temporal ordering or causality. Reverse associations and unmeasured factors, including depressive and broader internalizing symptoms, peer and family context, socioeconomic conditions, physical health, and access to physical activity opportunities, may partly account for the observed patterns. Second, all variables were assessed using self-report measures, which may introduce common method bias and reporting error. Moreover, the primary network was estimated using mean-centered, non-standardized scores from measures with different response ranges. Although the z-standardized sensitivity analysis yielded the same signs for all pairwise conditional associations, the symptom rumination–reflective pondering association, rather than the brooding–social interaction anxiety association, was the largest edge in the full standardized network. Thus, the absolute magnitudes and rankings of raw-score edge estimates are scale dependent and should be interpreted cautiously. The high internal consistency and conceptual overlap among the rumination subscales, together with the restricted four-construct network, may also have affected the allocation of shared variance across edge estimates and expected-influence rankings. No formal multicollinearity diagnostic, latent-variable analysis, or redundancy analysis was conducted; therefore, these possibilities could not be directly evaluated. Accordingly, the model should be interpreted as a focused construct-level network rather than a comprehensive representation of adolescent psychological functioning. Third, physical activity was assessed only using the PARS-3 composite exercise-volume score, which does not distinguish activity type, domain, social context, or degree of structure. Future studies should combine objective assessments, such as accelerometry, with domain-specific questionnaires or activity logs. Fourth, the CS coefficients for the interaction edges and interaction expected influence were both 0.28, indicating that the moderation findings remain preliminary and require independent replication. Finally, the sample was limited to adolescents aged 14–18 years from a single region of Shandong Province, China. Thus, the generalizability of the findings to other age groups, clinical samples, and regional or cultural contexts remains uncertain. Cultural norms concerning social evaluation, physical activity participation, and the expression of anxiety symptoms may influence both the levels of the study variables and the estimated network structure. Cross-cultural replication is therefore needed to examine the generalizability of these findings.

## 5. Conclusions

Based on a moderated network model, this study found that brooding showed the highest expected influence and the largest positive conditional association with social interaction anxiety among the three rumination dimensions. This finding suggests that brooding-related conditional associations may be a productive focus for future longitudinal research on rumination and social interaction anxiety. However, its high connectivity does not establish causal priority or confirm brooding as an intervention target.

Further results showed that several estimated conditional associations differed across physical activity levels, including the associations between symptom rumination and brooding, reflective pondering and brooding, and brooding and social interaction anxiety. However, because the data were cross-sectional and the interaction-related estimates showed limited stability, these moderation patterns should be regarded as exploratory. The findings do not establish that physical activity changed or reduced these associations. Longitudinal and experimental studies are needed to determine whether these patterns are reproducible and temporally meaningful.

## Figures and Tables

**Table 1:** Descriptive Statistics of the Study Variables.

Variable	M	SD	Range
Symptom rumination (A1)	18.10	7.20	12–48
Brooding (A2)	8.24	3.45	5–20
Reflective pondering (A3)	7.75	3.23	5–20
Social interaction anxiety (B1)	36.49	14.21	15–75
Physical activity level (M)	10.41	15.05	0–100

**Table 2:** Pairwise edge-weight estimates and bootstrap 95% confidence intervals.

Edge	Estimate	95% CI
Symptom rumination–brooding (A1–A2)	0.457	[0.361, 0.558]
Symptom rumination–reflective pondering (A1–A3)	0.821	[0.728, 0.911]
Symptom rumination–social interaction anxiety (A1–B1)	0.094	[−0.044, 0.227]
Reflective pondering–brooding (A3–A2)	0.386	[0.304, 0.464]
Brooding–social interaction anxiety (A2–B1)	1.197	[1.004, 1.420]
Reflective pondering–social interaction anxiety (A3–B1)	−0.108	[−0.388, 0.152]

## Data Availability

The datasets generated during and/or analyzed during the current study are available from the corresponding author on reasonable request.

## Supplementary Materials

The following supporting information can be downloaded at: https://www.mdpi.com/article/10.3390/bs16091461/s1, Figure S1: Expected influence values for symptom rumination (A1), brooding (A2), reflective pondering (A3), and social interaction anxiety (B1) in the primary moderated network. Figure S2: Nonparametric bootstrap accuracy plots for pairwise edge and expected influence estimates in the primary moderated network. Figure S3: Case-dropping bootstrap stability plots for pairwise edge and expected influence estimates. Table S1: Sensitivity analysis of conditional associations before and after adjustment for age and sex. Table S2: Scale-sensitivity analysis comparing pairwise conditional associations and expected influence between the primary raw-score and z-standardized moderated networks.

## Abbreviations

The following abbreviations are used in this manuscript:

RRS-CV	Chinese Version of the Ruminative Responses Scale
IAS	Interaction Anxiousness Scale
PARS-3	Physical Activity Rating Scale-3
EI	expected influence
CI	confidence interval
CS	correlation stability
