# Supplementary material for: Physical Activity, Rumination, and Social Interaction Anxiety in Adolescents: A Cross-Sectional Moderated Network Analysis
Source: Behav Sci (Basel). 2026 Aug 23;16(9):1461. doi: 10.3390/bs16091461 (PMC13603174; doi:10.3390/bs16091461)
Supplement: Supplementary file 1 [file behavsci-16-01461-s001.zip › behavsci-4415990-supplementary.pdf]

Supplementary Materials:

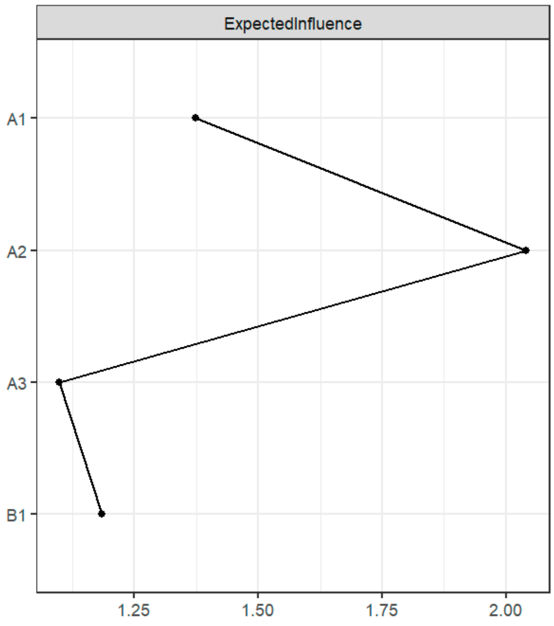

Figure S1. Expected influence values for symptom rumination (A1), brooding (A2), reflective pondering (A3), and social interaction anxiety (B1) in the primary moderated network.

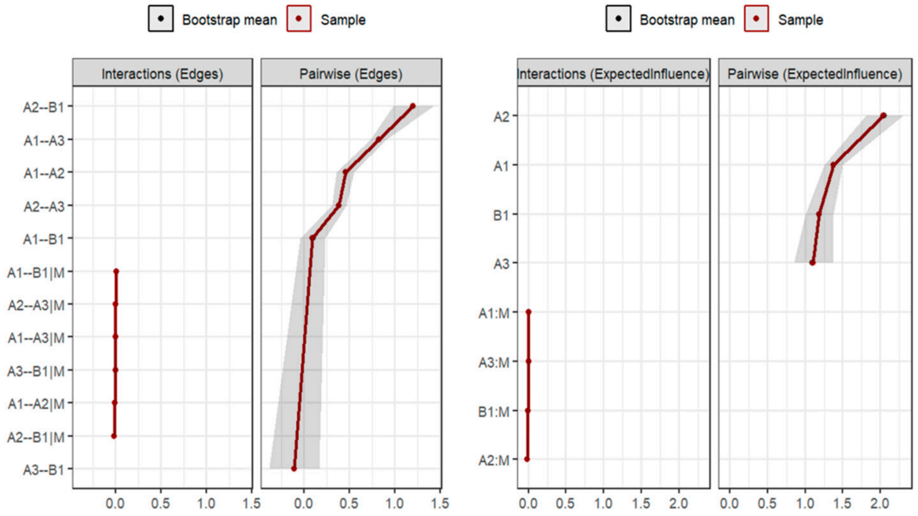

Figure S2. Nonparametric bootstrap accuracy plots for pairwise edge and expected influence estimates in the primary moderated network.

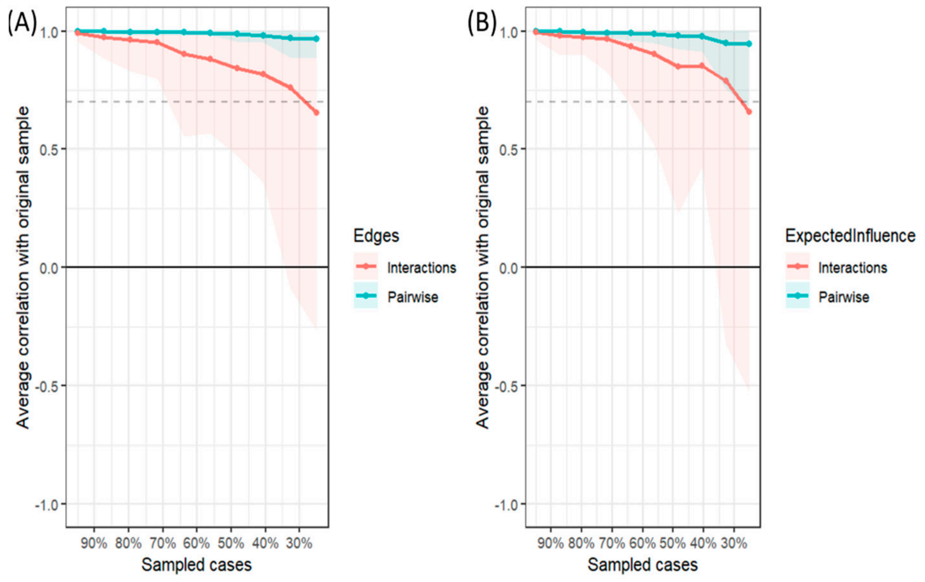

Figure S3. Case-dropping bootstrap stability plots for pairwise edge and expected influence estimates.

Table S1. Sensitivity analysis of conditional associations before and after adjustment for age and sex

Panel A. Pairwise conditional associations at the mean physical activity level

Association	Primary estimate	Primary 95% CI	Age- and sex-adjusted estimate	Adjusted 95% CI
A1-A2	0.457	[0.356, 0.551]	0.457	[0.355, 0.550]
A1-A3	0.821	[0.728, 0.911]	0.823	[0.730, 0.912]
A1-B1	0.094	[−0.044, 0.227]	0.080	[−0.052, 0.213]
A3-A2	0.386	[0.304, 0.464]	0.386	[0.303, 0.465]
A2-B1	1.197	[1.004, 1.420]	1.141	[0.947, 1.366]
A3-B1	−0.108	[−0.388, 0.152]	−0.014	[−0.280, 0.243]

Panel B. Max–min differences in the focal moderated associations before and after adjustment for age and sex

Moderated association	Primary max–min difference	Adjusted max–min difference	Primary 95% CI	Adjusted 95% CI
A1-A2	−0.205	−0.204	[−0.398, −0.004]	[−0.384, −0.001]
A3-A2	0.544	0.545	[0.156, 0.952]	[0.068, 0.969]
A2-B1	−3.571	−3.301	[−6.822, −0.509]	[−6.580, −0.336]

Table S2. Scale-sensitivity analysis comparing pairwise conditional associations and expected influence between the primary raw-score and z-standardized moderated networks

Panel A. Pairwise conditional associations at the mean physical activity level

Association	Primary raw-score estimate	Z-standardized estimate
A1-A2	0.457	0.385
A1-A3	0.821	0.618
A1-B1	0.094	0.055
A2-A3	0.386	0.382
A2-B1	1.197	0.353
A3-B1	-0.108	-0.029

Panel B. Expected influence at the mean physical activity level

Node	Primary raw-score EI	Z-standardized EI
A1	1.373	1.059
A2	2.040	1.120
A3	1.099	0.972
B1	1.184	0.379
